# Experimental and Numerical Assessment of Sustainable Concrete Using Recycled Concrete Powder (RCP) as a Partial Replacement for Cement

**DOI:** 10.3390/ma18133108

**Published:** 2025-07-01

**Authors:** Hafiz Asfandyar Ahmed, Waqas Arshad Tanoli

**Affiliations:** 1 Department of Construction and Quality Management, Hong Kong Metropolitan University, Hong Kong; engrasfandyar2009@gmail.com; 2 Department of Civil and Environmental Engineering, College of Engineering, King Faisal University (KFU), P.O. Box 380, Al-Hofuf 31982, Al-Ahsa, Saudi Arabia

**Keywords:** recycled concrete powder (RCP), global warming potential (GWP), microstructure, finite element analysis, dynamic modulus of elasticity

## Abstract

The demolition of structures generates waste that poses environmental, social, and economic challenges. This study explores the effects of incorporating recycled concrete powder (RCP) into concrete, using it as a cement substitute at levels of 0%, 20%, 25%, and 30%. We evaluated fresh properties like workability and hardened properties such as dry density, water absorption, compressive, flexural, and split tensile strength, along with non-destructive parameters and microstructural features. The study found that substituting 20% of cement with RCP does not significantly impact mechanical properties, while higher substitutions (25% and 30%) have a slightly greater effect. Notably, 20% RCP substitution resulted in a 15–18% reduction in compressive strength over 7 to 28 days. However, it also led to a 20% decrease in CO_2_ emissions. A numerical analysis using nonlinear finite element analysis for flexural beam simulations further validated these results. Overall, the study promotes sustainable concrete solutions, achieving a balance between strength, environmental impact, and eco-efficiency in construction.

## 1. Introduction

With growing development worldwide, abundant concrete demolition waste is produced [1,2]. Presently, the demolition waste generated annually in the European Union and the United States is nearly 800 and 700 megatons, respectively, and it crosses 1800 megatons in China [3,4,5]. The recycling percentage of demolition waste is above 70% in several developed countries like Japan, the United States, and Europe. However, it is presently less than 10% in China. Usually, tons of demolition waste are dumped in landfills [6,7]. About 300–400 kg/m^3^ of cement is used in concrete, and it uses 4–6 billion tons annually [8,9,10]. Carbon dioxide (CO_2_) released in concrete manufacture is about 80% due to the cement comprising 5–7% of total CO_2_ emissions [11].

Sustainable development is the main objective of today’s world. The growingly strict ecological guidelines and necessities of global treaties are recent developments, with major economies forming pertinent rules, regulations, and forthcoming strategic arrangements [12]. For instance, China has planned carbon peaking by 2030 and carbon neutrality by 2060. In the present circumstances, the construction field must stabilize the social, environmental, and economic phases to accomplish sustainable development [13]. Cement manufacturing vigorously needs substitute materials to attain sustainable development, green development, and a circular economy [14]. For example, cement factories contribute to air pollution by releasing solid waste [15]. These industries are responsible for significant emissions of greenhouse gases [16]. Addressing unnecessary energy use and the substantial CO_2_ emissions from the process is crucial to effectively manage and control cement manufacturing sustainably [17]. Moreover, appropriate waste disposal from several industries is a critical concern worldwide. The generation of waste from industries and inorganic by-products is on the rise due to expanding industrialization and the need for greater resources to maintain the rapidly expanding global population [18,19,20]. Therefore, properly handling unsafe by-products generated by various industries is becoming a significant issue, impacting energy supply and contributing to pollution. A substantial volume of waste material is generated each year due to building demolition for urban reconstruction on a global scale and is subsequently deposited in landfills [21,22]. Nevertheless, disposing of these wastes in landfills can harm the environment and compromise valuable land [2,21]. Consequently, the potential for recycling demolition waste in concrete production to minimize waste disposal has garnered significant attention [23]. The following sections will demonstrate that a partial replacement of cement with a small amount of recycled concrete powder (RCP) is a feasible and effective approach.

Researchers are increasingly directing their attention to nanotechnology, with nano-powders being utilized to enhance the properties and behavior of cementitious materials [24,25,26,27]. The incorporation of nanomaterials, like TiO_2_, Al_2_O_3_, and SiO_2_, into construction materials enhances their strength and workability characteristics [26,28]. Several significant findings in material behavior have had a profound impact on the development of nanoscale applications [29,30,31]. One of the current potential applications involves the utilization of RCP as a reactive constituent in cementitious materials [32]. Nanomaterials can serve as a filler or binder material to enhance the interfacial zone of the aggregates with the surrounding cement matrix [11,33], thereby enhancing the overall matrix quality [34,35]. Nanomaterials yield improved microstructure in the resulting products without altering their chemical composition [36].

During the demolition of concrete structures, a huge quantity of waste is produced [14], comprising a hydrated cement matrix and a small amount of un-hydrated C_2_S, C_3_S, CaO, and SiO_2_, which is hypothetically active [8]. Reusing and converting demolition waste into recycled concrete powder (RCP) for reuse in concrete is a superb aptitude. RCP demonstrates a specific level of pozzolanic reactivity, and increasing the fineness of ground-recycled concrete can enhance its pozzolanic reactivity [3]. Efficient use of RCP can replace cement, solve demolition waste issues, reduce CO2 emissions from concrete, and support sustainability, offering significant environmental and financial benefits [37,38]. The utilization of waste materials in construction offers numerous advantages. Primarily, it aids in reducing the volume of waste sent to landfills, thereby mitigating environmental pollution [39,40]. It helps conserve natural resources by repurposing waste into valuable materials. Using industrial waste in construction, like concrete and insulation, can significantly lower project costs [41], ultimately making housing and infrastructure more affordable. Utilizing waste in this manner can also foster the advancement of sustainable and environmentally affordable building practices that minimize the environmental impact [42,43]. In summary, converting waste into building material via effective recycling procedures offers a financially and ecologically viable solution for managing the escalating volume of waste [44,45]. This research uniquely converts waste into nanomaterials to study their effects on concrete performance. Its goals are to tackle waste management issues, explore sustainable solutions, and improve ecological sustainability in construction. Nano-recycled concrete powder (RCP) was added to concrete to evaluate its strength against control concrete with the same water-to-binder ratio, with no impact on hydration products or mechanical properties [46]. This research highlights the potential of using concrete waste materials, like RCP, as a partial substitute for cement in eco-friendly building materials. This approach can lower carbon footprints, reduce construction waste, and tackle environmental issues associated with these materials.

This study has developed a comprehensive experimental scheme; however, limitations in finances, time, and workforce restrict the number of samples that can be tested. Consequently, numerical investigation becomes essential, as it enables significant savings in time and resources. The Finite Element Method (FEM) is an effective approach for modeling structures composed of numerous elements, utilizing properties such as elastic modulus, tensile strength, and compressive strength. A key area of interest is the influence of heterogeneity on concrete fracture characteristics, which has been further clarified by advancements in numerical methods. Heterogeneity arises from factors such as voids, coarse aggregates, microcracks, unhydrated particles, and fibers. Further details regarding the numerical model and the characterization of concrete heterogeneity can be found in the work by Mukhtar and El-Tohfa [47]. It should be noted that the focus of the current study is to conduct an experimental study, and a numerical analysis is used to authenticate the results.

This paper explores RCP as a cement replacement to develop eco-friendly concrete without sacrificing strength or microstructure. The study examines RCP’s impact on mechanical, microstructural, and environmental characteristics, utilizing X-ray fluorescence and diffraction to analyze cement and RCP micro-properties. Various proportions of RCP were tested through theoretical and practical experiments to determine the optimal mix for strength, performance, and sustainability. Mechanical properties measured include compressive strength at 7, 14, and 28 days, flexural and split tensile strength at 28 days, water absorption, and dry density at 28 days. Static and dynamic elastic moduli were compared to understand concrete behavior under different loading conditions. Regression analysis correlated compressive strength with split tensile strength for RCP mixes. The influence of RCP on microstructural properties was assessed via scanning electron microscopy. Global warming potential was estimated in kg CO_2_, and volatile gas emissions per unit of concrete were calculated with the life cycle assessment (LCA) tool [48]. Finally, finite element analysis was performed with a four-point bending test to validate and optimize results among selected RCP proportions.

## 2. Research Significance

Incorporating recycled concrete powder (RCP) as a partial substitute for cement reduces the amount of cement used in concrete mixes. This substitution can improve the carbon footprint of concrete by minimizing cement use, which is responsible for significant CO2 emissions during production. Additionally, integrating RCP into concrete aligns with the utilization of construction and demolition materials. By reprocessing these materials as substitutes for cement, this paper aims to promote sustainability in the construction industry and provide solutions to the ecological challenges posed by construction and demolition waste.

This study’s aims are as follows:

- Address existing research gaps by investigating the use of recycled concrete powder (RCP) as a partial replacement for cement.
- Evaluate the potential of RCP to lower CO₂ emissions while maintaining the mechanical performance of concrete.
- Examine the effects of RCP on the mechanical and microstructural properties of concrete.
- Compare the dynamic and static elastic moduli in concrete mixes that include RCP.
- Develop a regression model that correlates compressive strength and split tensile strength.
- Utilize finite element analysis to gain insights into the structural behavior of concrete modified with RCP.

## 3. Experimental Work

**Figure 1:**
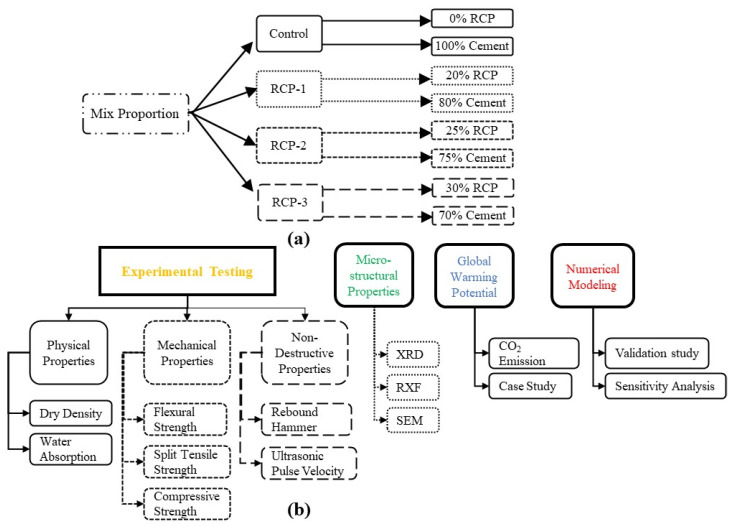
(**a**) Mix proportion (4 mixes considered); (**b**) scope of work containing experimental testing, microstructural properties, GWP, and numerical modeling.

The proposed research methodology, along with various mixed proportions considered in this study, is presented in Figure 1.

### 3.1. Materials

#### 3.1.1. Cement

Ordinary Portland cement (OPC) is readily accessible locally and was used to create the concrete mixtures. As per ASTM C-150 [49], it is type I cement. One of the limitations of this study is that only type I cement was considered.

#### 3.1.2. Recycled Concrete Powder (RCP)

**Figure 2:**
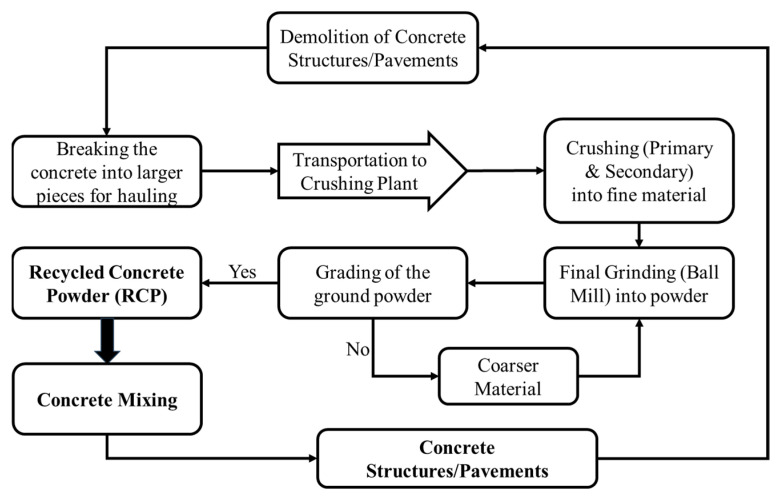
Manufacturing details and life cycle of recycled concrete powder (RCP).

**Figure 3:**
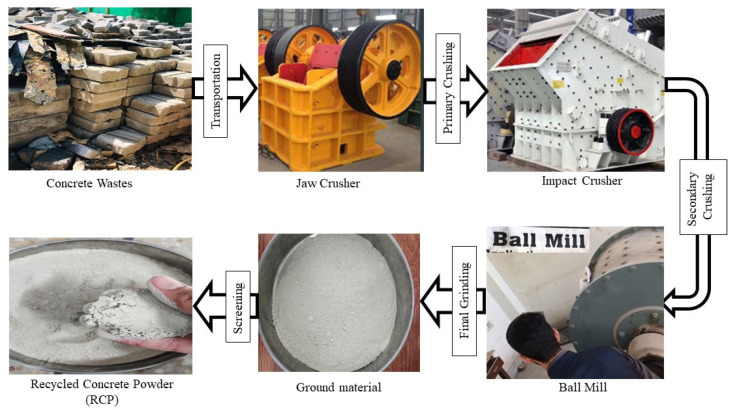
Various stages involved in the production of RCP.

Waste concrete was acquired from local construction waste in Peshawar, Pakistan, when it was ground and separated by a 0.025 in (0.63 mm) sieve and finally powdered thoroughly in a ball mill. Figure 2 presents the schematic details of various processes involved in the recycling of concrete waste into RCP, and Figure 3 presents various stages involved in the manufacturing of RCP. The average particle size of RCP after half an hour of ball mill processing is 0.0013 in (32.1 μm), and the average size of RCP particles after 1 h of ball mill processing is 0.0007 in (18.9 μm). The average particle size of cement is 0.0009 in (23.1 μm); therefore, the RCP with an average particle size of 0.0009 in (23.1 μm) after 1 h of ball mill processing is selected. The chemical properties of RCP are presented in Table 1.

#### 3.1.3. Characterization of Cement and RCP via XRF

**Figure 4:**
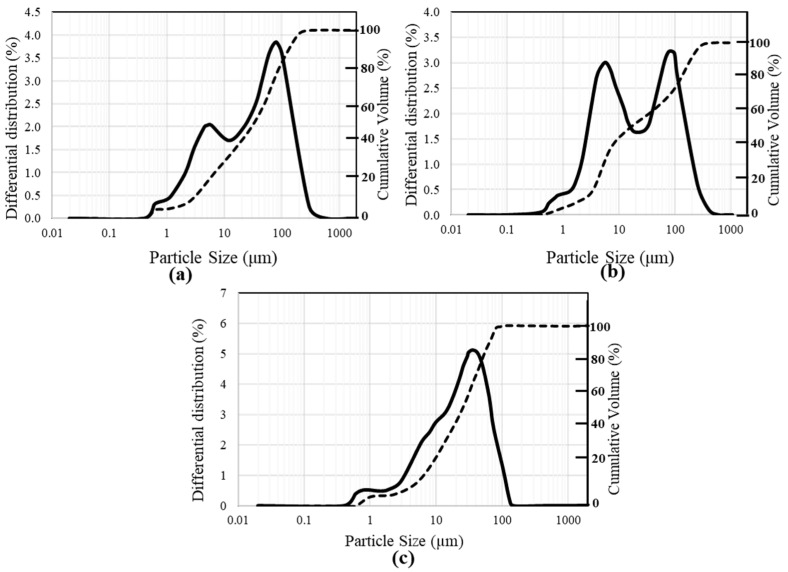
Particle size distribution of RCP at (**a**) 30 min and (**b**) 60 min ball mill processing, and (**c**) particle size distribution of cement.

The composition and chemical properties of cement are presented in Table 1. The chemical properties via XRF (X-ray fluorescence) indicate the presence of SiO_2_, Al_2_O_3_, and CaO as main oxide constituents in both cement and RCP (see Table 1); however, cement has 60.47% and 20.63%, while the RCP has 22.50% and 32.40% of SiO_2_ and CaO, respectively. The comparison of Figure 4a,b, and c reveals that the ratio of minute particles of size 1–10 μm of RCP surpasses cement ones, which offers the opportunity for RCP particles to plug the matrix and reduce the pores. In a nutshell, it can be stated that RCP is not as vigorous as cement, but it possesses some particle benefits. These results are, in general, coherent with a few modern studies [50,51,52].

#### 3.1.4. Characterization of Cement and RCP via XRD

**Figure 5:**
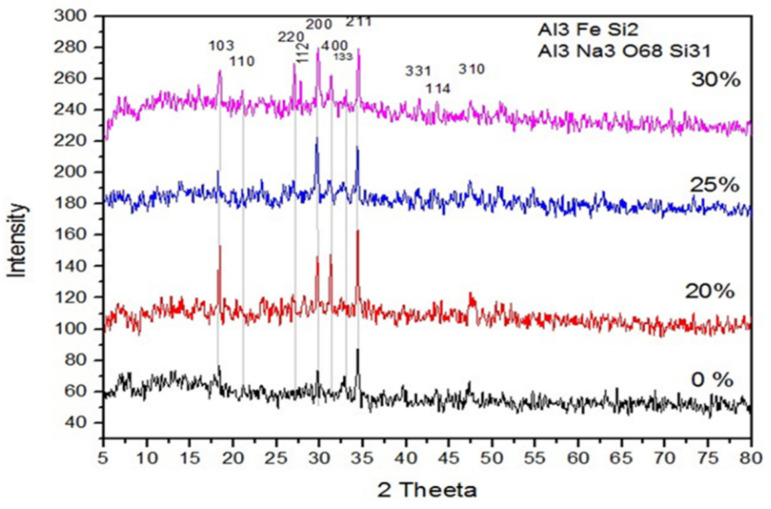
XRD test of cement and three replacements of RCP.

The X-ray diffraction (XRD) test for cement and all three replacements with RCP is depicted in Figure 5. XRD is a procedure used for studying materials at the molecular level by evaluating the X-ray scattering. This test specified the structure and composition of RCP, helping describe its properties and evaluate its appropriateness for many applications. As seen in Figure 5, several peaks of Al are observable in the XRD spectra. In the control sample, the analysis showed Centro-symmetrical, such as a large amount of Al (41.95%), Fe (28.94%), and Si (29.11%). But, in the binary mixes where RCP was used, the bond between the atom and particle is also centrosymmetric, but a large amount of amorphous content is found. The chemicals found in these mixes are Al_3_ Na_3_ O_68_ Si_31_, which shows that a large amount of oxygen and silicate is present due to the presence of pores and resulted in a weaker bond seen in SEM results. The XRD peaks refer to the less prominent of the crystalline phases of the cement matrix. This is due to the replacement of cement with amorphous RCP that does not exhibit a crystal structure. The absence of characteristic XRD peak intensity from the cement matrix would be an indication of the successful incorporation of the RCP in the cement matrix. Secondly, the XRD pattern would show broad peaks or humps resulting from the amorphous phases present within the mix. The pattern would not have any specific peak positions and would occur over a range of angles. This indicates that a significant amount of RCP has not fully reacted with the other components of the cement matrix. Thirdly, the absence of peaks corresponding to the crystalline phases that were present in traditional cement concrete also indicates that the partially replaced concrete mix has a different crystal structure as compared to the controlled sample.

### 3.2. Mix Proportions

**Figure 6:**
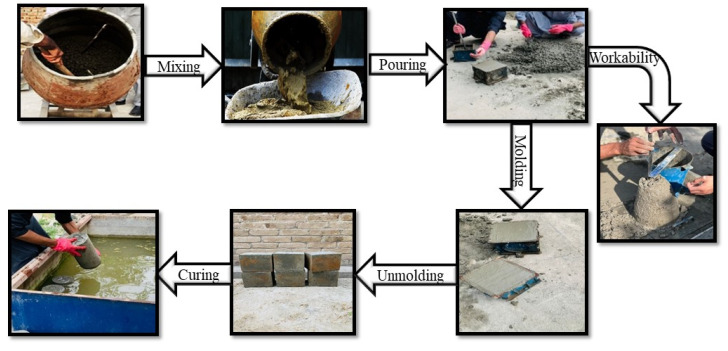
Steps involved in the preparation of concrete specimens.

Concrete constituents were mixed via a rotating mixer, as per ASTM C192 guidelines [53]. Various steps involving concrete mixing, specimen preparation, and curing are presented in Figure 6. Once the entailed slump was attained, the cylinder molds with a 150 mm diameter and 300 mm height, beam molds with the size of 100 × 100 × 400 mm^3^, and cube molds with the size of 150 × 150 × 150 mm^3^ were filled as per ASTM C39 [54]. Overall, 21 samples were made for every mix to evaluate its compressive strength at 7, 14, and 28 days of curing (three samples each), splitting tensile strength, and non-destructive testing at 28 days. The samples were taken out from the molds after twenty-four hours and set for curing until the testing age, i.e., 7, 14, and 28 days. The concrete specimens were capped before testing to make the surface level for uniform application of the load.

### 3.3. Testing Procedure

#### 3.3.1. Physical Properties

##### Water Absorption

A water absorption (WA) test was performed on all the concrete mixes as per ASTM C948 [55]. To conduct the WA test, concrete specimens were 28-day cured and were imbibed in water at 21 °C for 24 h and then intermittently weighed until the saturated-surface dry weight (SSD) steadied. The concrete specimen was oven-dried at 100−110 °C, and the weights were recorded after 24 h. Afterward, the specimen was placed in a vacuum desiccator to cool down at room temperature and weighed. To compute WA and AP (apparent porosity), Equations (1) and (2) were used:

(1) $$Water\;Absorption\;\left(\%\right)=\left(B-C\right)/C\times 100$$

(2) $$Apparent\;Porosity\;\left(\%\right)=\left(B-C\right)/\left(B-A\right)\times 100$$

where:

B is the SSD weight of the specimen.

A refers to the weight of the specimen suspended in water.

C refers to the weight of the oven-dried sample after it cools at room temperature.

##### Dry Density

The dry density of the binary mix of concrete specimens is performed as per ASTM C 642/06 [56]. Dry density refers to the density of a material without considering the presence of any moisture or water content. In the context of concrete, dry density represents the mass of solid concrete per unit volume, excluding the volume occupied by water.

#### 3.3.2. Mechanical Properties

The compressive and split tensile strength of concrete was evaluated following the ASTM C-39 [54] and ASTM C-496 [57], respectively. A minimum of five samples were tested for each type to lessen the experimental error.

#### 3.3.3. Non-Destructive Testing

Two of the non-destructive testing methods, i.e., the ultrasonic pulse velocity (UPV) method and the Rebound Hammer method, were employed as per ASTM C-597 [58] and ASTM C-805 [59], respectively. The UPV is a technique used to evaluate the quality and condition of concrete; however, in the rebound hammer test, the rebound number is a measure of the surface hardness of concrete. The results of these tests are associated with the mechanical properties of concrete, employing some in-depth calculations and investigations.

#### 3.3.4. Microstructural Behavior Using Scanning Electron Microscopy

The microscopic composition of the samples was examined using SEM JSM-5910 (JEOL Ltd., Tokyo, Japan), available at the University of Peshawar facility. SEM is a prevailing imaging method that applies a focused electron beam to generate a clear picture of the specimen’s surface. It provides a detailed interpretation of the specimens’ surface traits, morphology, and composition.

### 3.4. Global Warming Potential (GWP)

The GWP of various mixtures was estimated in terms of CO_2_ (kg CO_2_-eq) employing the LCA tool, which is particularly intended to estimate the impact of concrete on the environment by considering its various basic materials and utilization of water and fuels. The CO_2_ emission of the four mixes opted in the current research was calculated [48]. Various assumptions are made for different manufacturing technologies, physical locations, distances, methods of transport, electricity power grids, and types of materials, are taken from [60].

## 4. Numerical Modeling

### 4.1. Concrete Damage Plasticity

**Figure 7:**
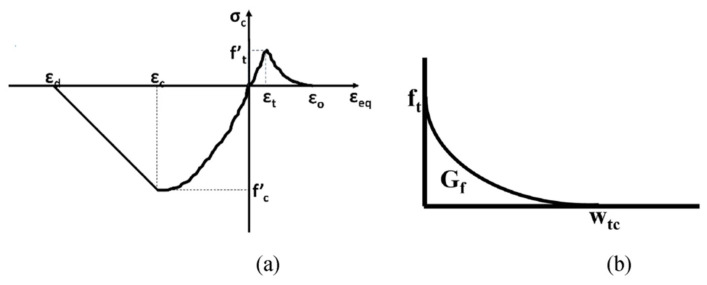
(**a**) Stress–strain curve and (**b**) tensile softening of concrete.

The “3D-Nonlinear Cementitious2” material model available in the Atena 3D V.5 [61] was used to model the concrete. This model is chosen for its computational proficiency and accuracy in representing local responses and failure patterns. It is designed to estimate the behavior of concrete and similar materials, such as masonry and rock. The main failure types described in the model are tensile cracking and compressive crushing. It effectively captures the damage caused by compressive and tensile stresses through both micro and macro cracks. Parabolic and exponential laws are used to define the compressive hardening and tensile softening properties of concrete; see Figure 7. The material properties (i.e., f’_c_, f_t_, etc.) opted for the various model descriptions, which were calculated experimentally, are given in Table 2. It is important to note that the poison ratio used in this study was obtained from the literature rather than determined experimentally (Section 3 provides details of experimental testing, and Section 5 provides the results of experimental testing). Moreover, the values of E and GF were automatically calculated by the finite element software.

### 4.2. Geometry and Meshing

**Figure 8:**
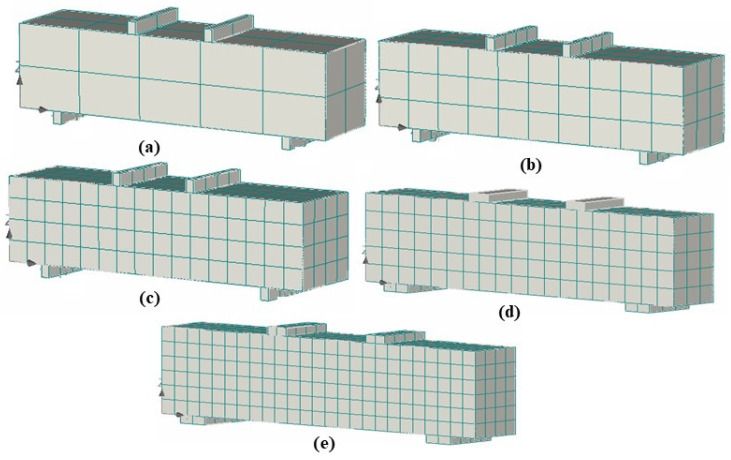
Mesh sensitivity analysis with mesh size of (**a**) 50 mm, (**b**) 34 mm, (**c**) 25 mm, (**d**) 20 mm, and (**e**) 17 mm.

It is generally believed that the finer mesh provides more accurate results of cracks and other stress concentrations, leading to more accurate detailing. Nevertheless, finer meshes may not necessarily yield accurate results when modeling brittle materials [31,62,63,64]. Generating many elements can lead to problems, specifically numerical uncertainty, in the cracking process. Therefore, a mesh sensitivity analysis was conducted (see Figure 8) to determine the most suitable mesh size for the test assembly involving a concrete beam and steel plates (for load application and support); several factors were considered, including computational time and accuracy in result predictions. After careful evaluation, a mesh size of 25 mm was deemed optimal (based on computation time and accuracy of results, among other important parameters) and was used for all analyses in this study.

### 4.3. Loading and Boundary Conditions

**Figure 9:**
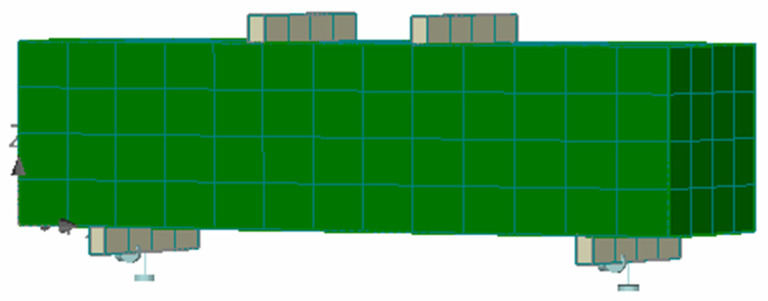
Meshing details, along with loading and support plates.

Figure 9 presents the specimen geometry, optimized mesh size, and loading details. The beam size was 400 × 100 × 100 mm, and steel plates of 12 mm width were used at the top and bottom of the specimen. A load control environment was used to test the models by employing a specified load on the steel plates at the top. The load was applied to the top plate, and the bottom plate was fixed to match the experimental conditions. An eight-node 3D solid brick isoparametric element, integrated by Gauss integration, was chosen for concrete modeling. Linear interpolation is used with a 4 × 4 Gauss integration system. Moreover, the fixed crack model (already available in the software) was used to govern the post-peak behavior of the material.

## 5. Results and Discussion

As mentioned in Section 3, a minimum of 3 samples were used for each mix to conduct the test, and then an average of those values was used in the graphs presented in this section.

### 5.1. Fresh Properties

#### 5.1.1. Standard Consistency

**Figure 10:**
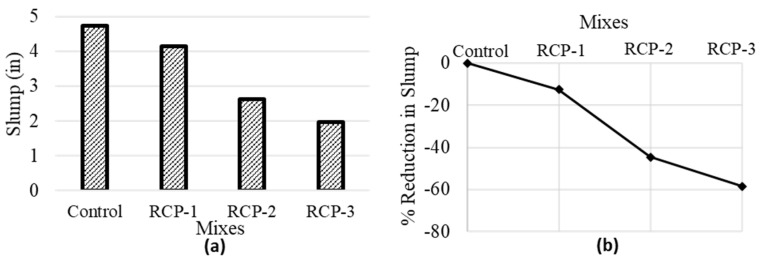
Average values of consistency test (**a**) slump values and (**b**) % reduction in a slump for all concrete mixes.

The standard consistency (SC) of all four mixes is shown in Figure 10. The test results indicate that the SC of mixes follows a declining pattern by increasing the RCP content in the mix. The SC is almost unaffected when the RCP replacement amount is less than 10%. The SC reduces by 15%, 45%, and 60%, in the case of RCP-1, RCP-2, and RCP-3, respectively. The angular shape of RCP particles and irregularity are significant parameters for escalating water requirements [65]. Many researchers consider the loss of ignition and larger surface area of RCP that causes a greater amount of water required by RCP particles, causing lowered fluidity [3].

#### 5.1.2. Dry Density

**Figure 11:**
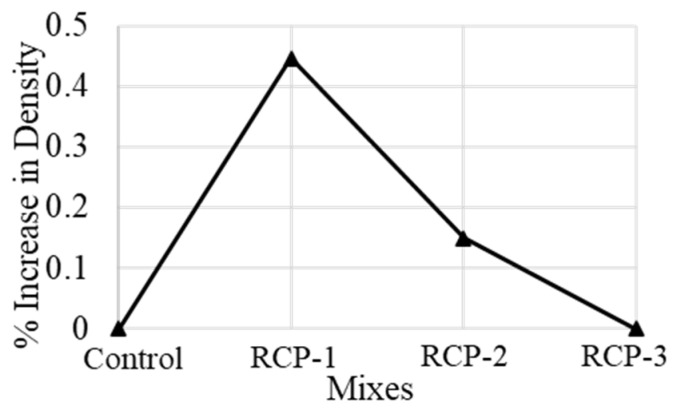
Percentage increase in dry density for all the concrete mixes.

Figure 11 presents the dry density values for various concrete mixes and the % increase with the inclusion of RCP as a cement substitute. It can be seen that all the concrete mixes possess almost the same amount of dry density, and there is only a 0.45% difference between various mixes, which is negligible and can be attributed to human error or test error.

#### 5.1.3. Water Absorption

**Figure 12:**
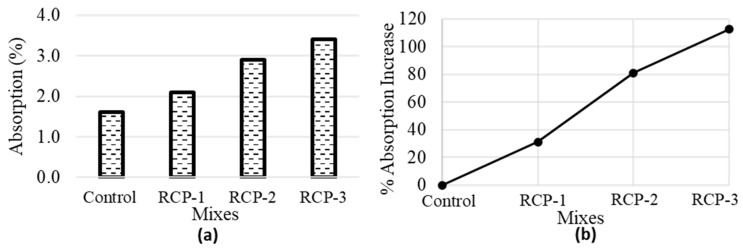
Average values of (**a**) water absorption and (**b**) % increase in absorption for all concrete mixes.

Figure 12a presents the values of water absorption for the control mix in comparison with the RCP mixes. The % increase in water absorption is also presented in Figure 12b, which indicates that the water absorption increases by 30%, 80%, and 112% for RCP-1, RCP-2, and RCP-3 mixes, respectively. RCP has a greater water demand as compared to cement, and by increasing the RCP content in the mix, the absorption of water also rises, and the resulting water-to-binder ratio drops, reducing the setting time of the mix. Nevertheless, with the RCP quantity growing, the water absorption rises in the RCP mixes, resulting in the fluidity reduction in the mix [66].

### 5.2. Mechanical Properties

#### 5.2.1. Compressive Strength

**Figure 13:**
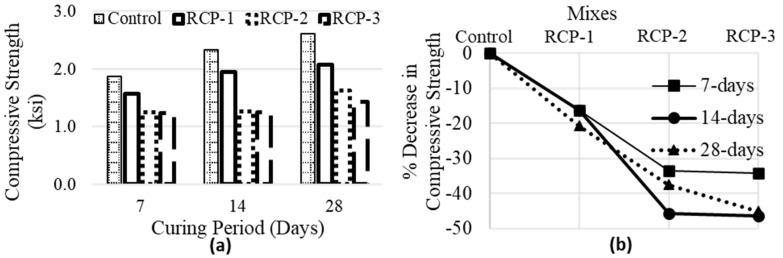
Average values of (**a**) compressive strength and (**b**) % reduction in compressive strength for all concrete mixes.

The impact of different RCP contents on the compressive strength (CS) of concrete is presented in Figure 13. By increasing the RCP contents, the CS of concrete at the same age for all replacement percentages indicates a declining pattern. Figure 13b presents the % reduction in compressive strength of each mix in comparison with the control mix.

For the RCP-1, the CS (taken as f’_c_ in various codes) decreased relative to the control mix. At 7 days, the CS was 1567.91 psi (12.9 MPa), representing a decrease of approximately 16.2% relative to the control mix. At 14 days and 28 days, the strength values were 1951.15 psi (16.07 MPa) and 2070.83 psi (18.01 MPa), respectively, showing reductions of around 16.3% and 20.7%. In the case of the RCP-2, the CS decreased further. At 7 days, the strength measured 1243.63 psi (8.57 MPa), exhibiting a reduction of approximately 33.5%. At 14 days and 28 days, the strength values were 1266.16 psi (8.73 MPa) and 1633.39 psi (11.3 MPa), respectively, indicating decreases of around 45.7% and 37.5% compared to the controlled sample. The highest RCP content, RCP-3, resulted in the largest decrease in CS. At 7 days, the CS was 1229.92 psi (8.48 MPa), reflecting a decrease of approximately 34.3% compared to the controlled sample. At 14 days and 28 days, the strength values were 1250.99 psi (8.63 MPa) and 1434.19 psi (9.89 MPa), respectively, showing reductions of around 46.3% and 45.1% compared to the control sample. The decrease in compressive strength observed in samples with varying amounts of RCP can be attributed to several factors influenced by the chemical alignment of cement and RCP. Recalling the findings of the chemical analysis presented in Section 3.1 of this study, the following reasons can be considered. The chemical composition of RCP differs considerably from cement, and the presence of higher percentages of elements such as SiO_2_, Al_2_O_3_, and Fe_2_O_3_ in RCP compared to cement can affect the chemical reactions during hydration. These elements may have a diluting effect on the cementitious properties of the mix, which is important to a decrease in strength. Another reason is that cement contains a higher amount of calcium oxide (CaO), which plays a crucial role in forming the CSH gel and is responsible for the strength gain in concrete.

#### 5.2.2. Static Modulus of Elasticity

**Figure 14:**
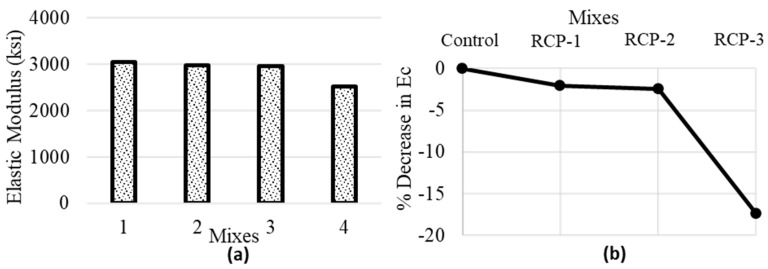
Average values of (**a**) modulus of elasticity and (**b**) % reduction in modulus of elasticity for all concrete mixes.

The modulus of elasticity (E_c_) of concrete is a vital feature in assessing the structural behavior and response of concrete elements (see Figure 14). Understanding the E_c_ helps in predicting the flexural deflection, cracking, and overall behavior of concrete structures under various loading conditions. This knowledge is crucial for ensuring the safety, durability, and functionality of concrete elements in construction projects. Researchers have attempted to estimate the static modulus of elasticity of concrete with the basic CS test, and they have succeeded. Since new concrete types are formed, and hence new formulations have been offered to simulate them. Table 3 summarizes the details of the models used to determine the E_c_ values based on f′_c_ primarily and other factors like constants and coefficients if used. In Noguchi’s model, k1 = 1, k2 = 0.956 are taken in this study.

Table 3 indicates that while following any of the models, the elastic modulus of RCP mixes does not differ much as compared to the control sample, especially RCP-1, which shows the E_c_ values close to the control mix, thus making it a very suitable replacement option.

#### 5.2.3. Split Tensile Strength

**Figure 15:**
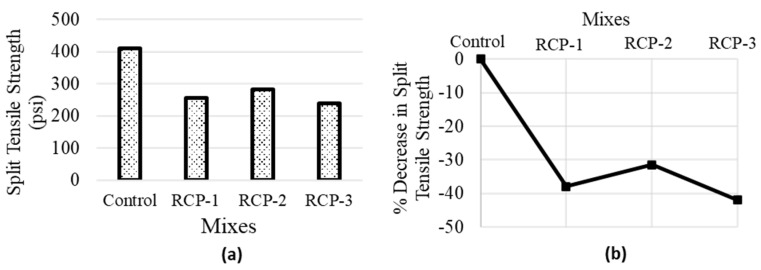
Average values of (**a**) split tensile strength and (**b**) % decrease in split tensile strength of all concrete mixes.

A split tensile strength test is performed on concrete cylinders following ASTM C496 [57]. The results showed interesting findings and are presented in Figure 15. Firstly, evaluating the percent decrease in strength in comparison with the control sample. When 20% of RCP was used as cement replacement in concrete, the split tensile strength decreased to 255.3 psi (1.76 MPa), which represents a decrease of 38% compared to the control sample, whose strength was 410.9 psi (2.83 MPa). This reduction in strength can be credited to the existence of RCP, which negatively affected the bonding and interlocking properties of the mix. Surprisingly, the split tensile strength increased in the case of RCP-2 as compared to RCP-3; however, it was the only case where RCP-2 showed superior performance, which can be attributed to some kind of test error. The strength at this percentage increases to 281.83 psi (1.94 MPa), which is approximately 31% less than the control sample. In RCP-3, the split tensile strength dropped to 238.64 psi (1.65MPa), which signifies a reduction of around 42% compared to the control sample. This decrease could be because of the excessive amount of RCP present in the mix, which might have badly affected the workability and cohesiveness of the mix. The higher percentage of RCP contents might have deferred the proper packing of aggregates and caused a less dense and weaker mix.

It can be concluded that the RCP differs from cement in chemical composition, and the presence of higher percentages of elements such as SiO_2_, Al_2_O_3_, and Fe_2_O_3_ in RCP can affect the chemical reaction process in the hydration reaction. These elements may have a diluting effect on the cementitious properties of the mix, leading to a reduction in strength.

#### 5.2.4. Correlation of Compressive Strength with Split Tensile Strength

**Figure 16:**
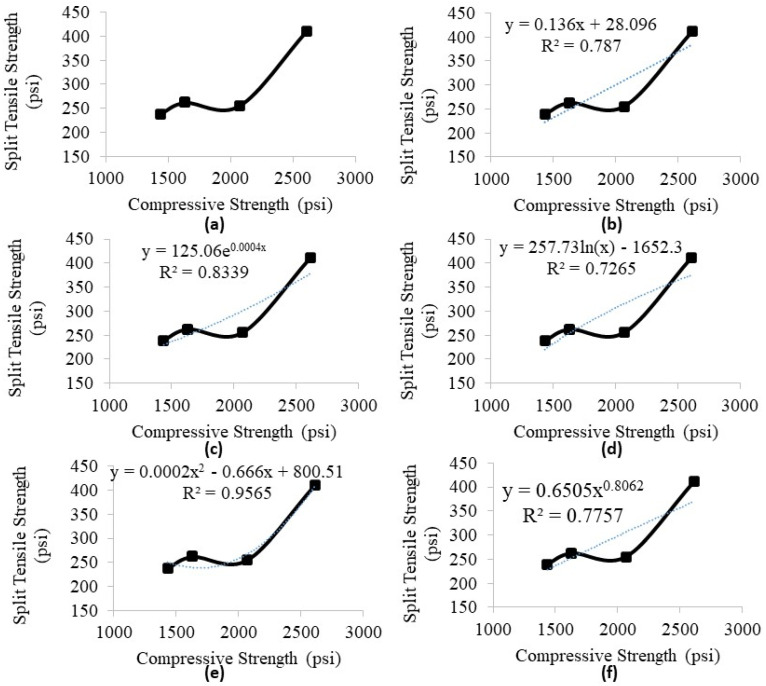
Regression analysis for determining the correlation of compressive strength with split tensile strength (**a**) original, (**b**) linear curve, (**c**) exponential curve, (**d**) logarithmic curve, (**e**) polynomial curve, and (**f**) power curve.

The association of the CS with split tensile strength for various RCP proportions is explored in this section. A regression analysis is performed to determine the best-fit curve. Figure 16 presents the results of the regression analysis. Based on the results, the polynomial relation (given by Equation (3) below) best fits the results obtained in this study. Thus, the quality-suit line is as follows:

(3) $$\;y=0.0002x^{2}-0.666x+800.51$$

where x is the CS (psi), y is the split tensile strength of concrete (psi), and R^2^ = 0.9565.

In Figure 16, it is evident that the line match showed a robust relationship, and the deviations were large compared to the results. Thus, it is possible to infer the precision of determining the CS of the concrete with RCP through a split tensile strength test and vice versa, signified by the R^2^ value. The split tensile strength tends to decrease when the CS decreases and vice versa. It should be noted that similar data were used in Figure 16 for all the curves shown.

### 5.3. Non-Destructive Testing

#### 5.3.1. Surface Hardness

**Figure 17:**
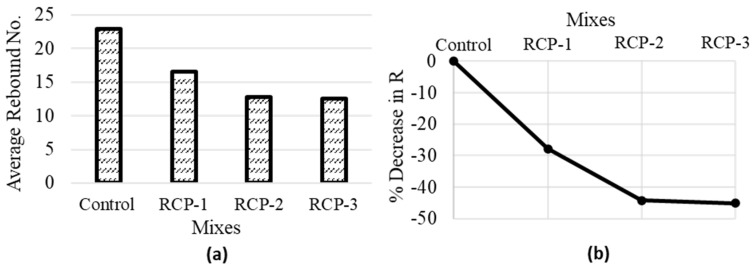
Average values of (**a**) Rebound No. (R), and (**b**) % decrease in rebound No. for all concrete mixes.

The rebound number is a measure of the hardness or stiffness of concrete. According to ASTM C 805 standards [59], a higher rebound number indicates better quality and higher stiffness of concrete. Table 2 shows that RCP contains a higher percentage of SiO_2_, Al_2_O_3_, Fe_2_O_3_, and CaO compared to cement. These elements result in the strength and hardness of concrete. However, RCP also contains a notably higher fraction of loss on ignition, representing a higher quantity of combustible or organic constituents. The existence of added constituents in RCP can result in a slightly lower rebound number compared to the control group. This could be due to differences in the properties and composition of RCP compared to cement. The higher loss on ignition in RCP may also cause reduced stiffness and hardness, resulting in lower rebound numbers. Furthermore, the results indicate that the average rebound number of RCP mixes decreases as the replacement fractions increase (see Figure 17).

#### 5.3.2. Ultrasonic Pulse Velocity

According to ASTM C597 [58], the results of UPV tests are presented in Table 4. The control samples showed higher pulse velocities in comparison with specimens with varying percentages of RCP. The control sample had a pulse velocity of 4.64 km/s, while RCP1, RCP2, and RCP3 had pulse velocities of 4.52, 4.24, and 4.35 km/s, respectively. Lower velocity values indicate the presence of cracks and reduced concrete quality. In this case, the reduction in pulse velocity in the case of RCP mixes can be related to various factors, including the chemical composition of the RCP.

#### 5.3.3. Dynamic Modulus of Elasticity

The dynamic modulus of elasticity (E_d_), which can be obtained from the UPV test results, assesses the material’s ability to resist deformation due to the dynamic loading conditions. In addition to that, the stiffness and strength parameters of the material can also be calculated and are very useful in determining the quality of the material. A larger value of E_d_ refers to a stiffer and more robust material, whereas a smaller E_d_ denotes a relatively more flexible and less resistant material. In terms of comparison with E_c_, E_d_ is generally greater for most of the materials, including concrete. It can be because of the nature of dynamic loading that induces more energy into the material, making it behave relatively stiffer as compared to the static loading conditions.

ASTM C 597 [58] provides a formula that uses pulse velocity, Poisson’s ratio, and concrete density to calculate the E_d_ of the concrete. Equation (4) is as follows:

(4) $$E_{d}=\rho v^{2}\frac{\left(1+\mu )(1-2\mu \right)}{\left(1-\mu \right)}$$

where ρ is the concrete density, and μ is Poisson’s ratio, 0.2 per ACI-318 [72].

**Figure 18:**
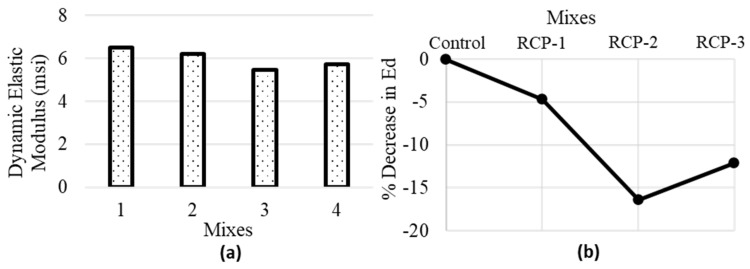
Average values of (**a**) dynamic modulus of elasticity and (**b**) % reduction in dynamic modulus of elasticity for all concrete mixes.

The Ed values obtained from Equation (4) for various concrete mixes and the % decrease in E_d_ are presented in Figure 18. The determination of E_d_ by this method involves the use of pulse velocity, as discussed in Section 4.3. The pulse velocity obtained for RCP-2 was less than that of RCP-3; therefore, the E_d_ value of RCP-2 is lower compared to RCP-3.

#### 5.3.4. Correlation of Dynamic and Static Elastic Moduli

After performing UPV tests on concrete specimens, a compressive strength test was conducted on them, the results of which are already discussed in Section 5.2.1. From those CS values, the E_c_ was determined and also discussed in Section 5.2.2. Similarly, the E_d_ determined from the UPV test is discussed in Section 5.3.2. This section discusses the correlation between E_d_ and E_c_. The E_d_ represents the elastic properties of a material under dynamic or cyclic loading, while the E_c_ represents the elastic properties under static loading.

Numerous equations related to E_d_ and E_c_, e.g., Lydon and Balendran [73], offered the following relationship between E_d_ and E_c_:

(5) $$E_{c}=0.83E_{d}$$

$$\mathrm{Or}\;\frac{E_{c}}{E_{d}}=0.83$$

Since five different models were utilized to determine the static elastic modulus (E_c_), by referring to Equation (5), we can identify which model in Table 3 effectively predicted the E_c_. According to ASTM C597 [58], the results of UPV are presented in Table 4. The control samples showed higher pulse velocities as compared to the mixes with various RCP proportions. The control sample had a pulse velocity of 4.64 km/s, while RCP-1, RCP-2, and RCP-3 had pulse velocities of 4.52, 4.24, and 4.35 km/s, respectively. The smaller velocity values indicate the crack’s presence and the lower quality of the concrete. In this case, the reduction in pulse velocity for RCP mixes can be related to the chemical composition of the RCP.

Table 5 displays the E_c_ to E_d_ ratio, and as E_c_ was determined using five different models, each model is employed to identify the most suitable one based on the ratio estimated by Equation (5). The findings reveal that the model proposed by Danha et al. [68] for determining the E_c_ of concrete from f^’^_c_ yields relatively better results, with the ratios for various concrete mixes closely aligning with the value of Equation (5).

The BS8110 Part 2 [74] suggests another equation for the estimation of E_c_ as follows:

(6) $$E_{c}=1.25E_{d}-19$$

Equation (6) allows for determining E_c_ values from E_d_ and vice versa. The E_d_ values outlined in Section 4.3 are employed in Equation (6) to ascertain the values of E_c_. Subsequently, the obtained E_c_ values are divided by E_d_ to verify the E_c_/E_d_ ratio using Equation (5).

For the control, RCP-1, RCP-2, and RCP-3 mixes, the E_c_ values derived from Equation (6) are 5393.70, 5012.07, 4059.03, and 4406.63, respectively. The corresponding E_c_/E_d_ values obtained are 0.83, 0.81, 0.74, and 0.77 for the control, RCP-1, RCP-2, and RCP-3 mixes, demonstrating close agreement with the value of Equation (5). Consequently, the dynamic and static elastic moduli are interrelated and reflect various performance parameters of the material. When considering the addition of new materials to concrete, such as RCP in this case, it is imperative to investigate their impact on various properties of concrete to comprehensively understand the behavior of the new mix and the impact of additional materials on the molecular properties of concrete.

**Figure 19:**
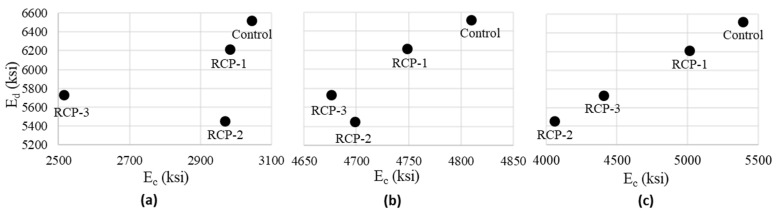
Dynamic vs. static modulus of elasticity for all concrete mixes using E_c_ from (**a**) ACI-363.R model, (**b**) Danha et al., 2013 [68] model, and (**c**) BS8100 model.

The ratio E_d_/E_c_ plays a crucial role in indicating material damping and energy dissipation capabilities. Therefore, it is imperative to comprehend the impact of cement replacement on these parameters of structural concrete. Figure 19 presents the correlation of E_d_ with the E_c_. The E_c_ derived from three different models is plotted against the E_d_ to better understand the efficiency of these models and the correlation between E_c_ and E_d_. To provide a clear overview, average values for each mix have been plotted. Notably, Figure 19b,c exhibit a more pronounced relationship between E_c_ and E_d_ in comparison to Figure 19a. Consequently, the determination of these parameters is of utmost importance and demands careful consideration based on experimentally evident results. However, it is essential to recognize that E_c_ and E_d_ are interdependent and can be derived from each other to meet specific usage requirements.

### 5.4. Analysis of Microstructure

**Figure 20:**
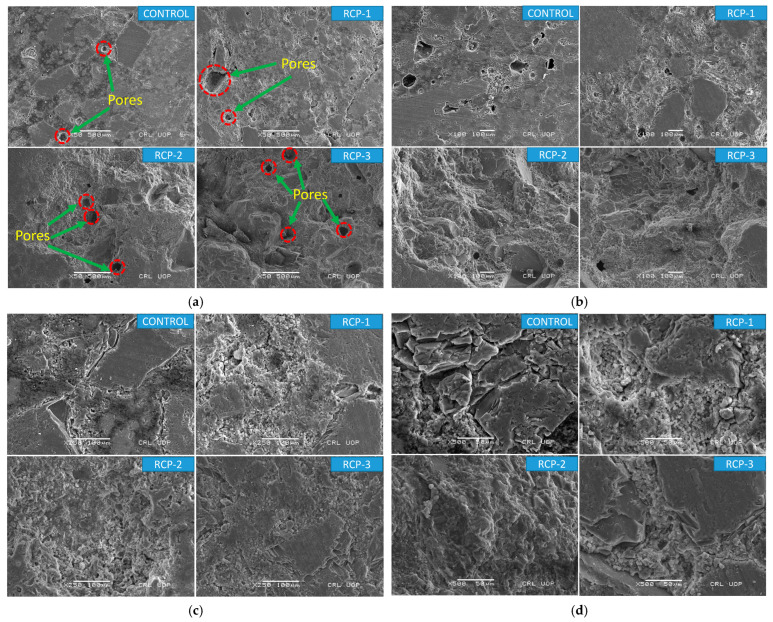

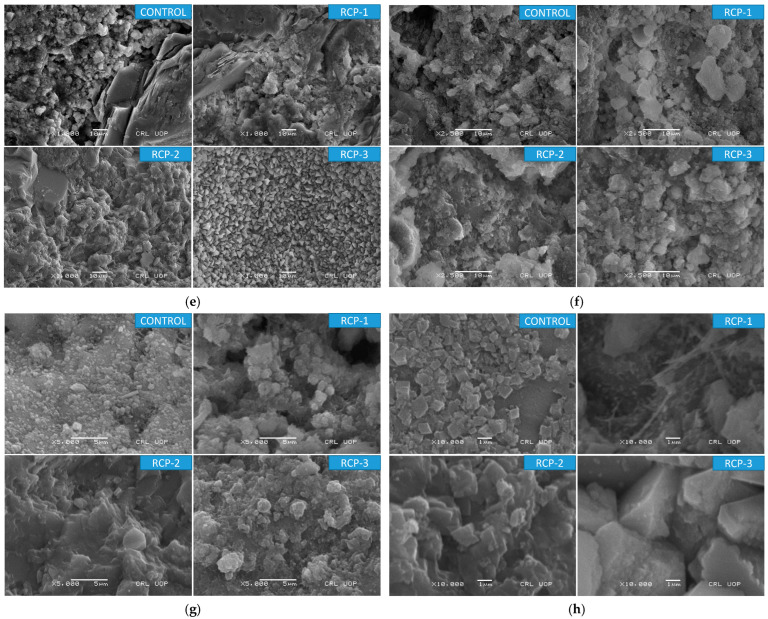
SEM images of all considered concrete mixes at a magnification of (**a**) 50×, (**b**) 100×, (**c**) 250×, (**d**) 500×, (**e**) 1000×, (**f**) 2500×, (**g**) 5000×, and (**h**) 10,000×.

To further investigate the reasons for obtaining the above-mentioned results, an in-depth investigation of the internal structure of concrete mixes was conducted. The SEM images of the concrete mixes are presented in Figure 20a–h, with various magnification levels from 50× to 10,000×, respectively. As predicted from the published literature, it was observed that the controlled mix yields a large quantity of CSH gel and CH as compared to the RCP mixes. By increasing the amount of RCP, the CSH gel formation tends to decrease, fostering the results mentioned in Section 4.2 and Section 4.3 of this study. In RCP-1, it is noticeable that the CSH gel reduces, and the CH is not ample. In RCP-2, the CSH further decreases; whereas there was not ample CH, certain fine RCP particles emerged in the mix at the same time. The hydration products in a controlled mix after 28 days produce better CSH gels. However, the CSH gel and CH quantity in the RCP-3 is considerably less, and the amount of fine RCP particles is significantly more. Figure 20 presents the results of the SEM test with different magnification levels. The irregular shape and morphology of the control sample are obvious, yet a strong CSH bond is present, resulting in strong particle binding and enhanced properties and lifespan of the concrete. The image reveals the presence of small pores, which could potentially compromise the structure if attacked by organic compounds, weakening the particle bond and affecting its properties. Like the control sample, the irregular shape and morphology of RCP-1 are apparent, yet the RCP particles are larger, potentially affecting the hydration process and dropping the concrete’s workability.

Moreover, it is revealed that weak CSH bonding between the particles and the presence of a substantial number of pores in the RCP-1 influence the concrete’s fresh and hardened properties. The morphology of RCP-2 reveals irregularly shaped particles and a significant surface area of RCP. Although numerous weak C-S-H bonds are present, an excessive number of pores may affect the permeability of concrete and thus result in increased water absorption of the mix.

A few distinct features can be described; firstly, SEM results indicate that the surface of RCP mixes appears rougher and more irregular compared to the control mix. Secondly, it is estimated that the microstructure of the concrete becomes more porous and permeable by the inclusion of RCP. Pores are formed due to the reaction between the RCP and the other constituents of the concrete. Moreover, the presence of C-S-H gels and other hydration products is also shown by the SEM results. Thirdly, depending on the nature of the RCP, it also reveals the presence of new phases in the concrete. These new phases include amorphous materials, different types of hydrates and oxides, and other secondary minerals formed as a result of the reaction.

The purpose of the above-mentioned results can be that the RCP acts with the CH present in the cement portion to produce CSH gels; similarly, the fine RCP fragments offer extra places to encourage the production of RCP. Additionally, by increasing the quantity of RCP, the cement is reduced by the same amount as the mix, resulting in a reduction in the generation of CSH and CH. Alternatively, the RCP also utilizes a portion of the CH, and a huge quantity of RCP particles are scattered near the hydration products. Many studies with similar descriptions illustrate that these products made through the initial hydration of RCP cause poor action, adversely influencing the RCP concrete’s CS [8,75]. The substitution quantity of RCP to cement, around 20%, has the least impact on the properties of the concrete mix [76,77,78].

### 5.5. Global Warming Potential

**Figure 21:**
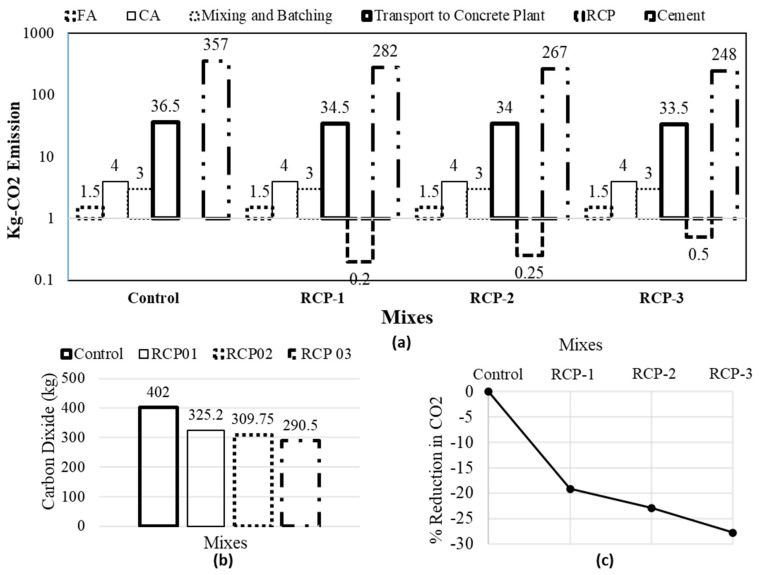
CO_2_ emission details for (**a**) various ingredients used, (**b**) overall CO_2_, and (**c**) % reduction in overall CO_2_ for all concrete mixes.

In Figure 21, the estimated GWP in kg CO_2_-equivalent per concrete volume is compared among the mixes. The figure illustrates the distribution of CO_2_-equivalent for each concrete mixture based on its constituents and key manufacturing procedures. The details of the inputs used in the calculation process are presented in [60]. The total CO_2_ emissions for each mix were estimated by considering the direct emissions and the emissions related to mining, manufacturing, and transport methods in the system’s boundary.

From Figure 21, it is evident that cement is responsible for the highest CO_2_ emissions as compared to other ingredients and processes considered in the manufacturing process of concrete. After cement, the materials transport shows the next-highest CO_2_ emissions source, ranging between 9.1% and 11.5%. Notably, the emission of CO_2_ related to non-cementitious materials is minimal and remains consistent across all concrete mixes. The RCP constituted only 0.06% to 0.2% of the total CO_2_ produced in the process. Likewise, the mixing and batching of concrete comprise small percentages, from 0.7% to 1%.

Based on the comparison, it was observed that the control sample demonstrated the highest GWP at 402 kg CO_2_ among all the mixes (Figure 11). Conversely, the RCP-3 exhibited the lowest GWP at 290.5 kg CO_2_. Overall, reducing the quantity of cement in concrete and increasing the RCP contents resulted in a respective 20%, 24%, and 29% reduction in the CO_2_ emission for RCP-1, RCP-2, and RCP-3.

**Figure 22:**
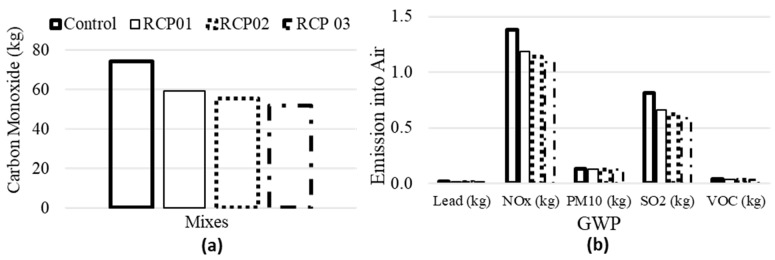
Emission of (**a**) carbon monoxide and (**b**) other volatile gases for all concrete mixes.

Similarly, the impact of RCP on other volatile gas emissions is also considered. Figure 22a presents the emission of carbon monoxide, and Figure 22b presents the emission of lead, NOx, PM_10_, SO_2_, and VOC for all the considered four concrete mixes. It can be seen that a similar pattern is observed, as presented in Figure 21 and discussed above. Thus, the inclusion of RCP in concrete decreases the demand for cement without significant reduction in its mechanical properties and increases sustainability by reducing CO_2_ and other volatile gas emissions.

## 6. FE Model, Results, and Discussion

**Figure 23:**
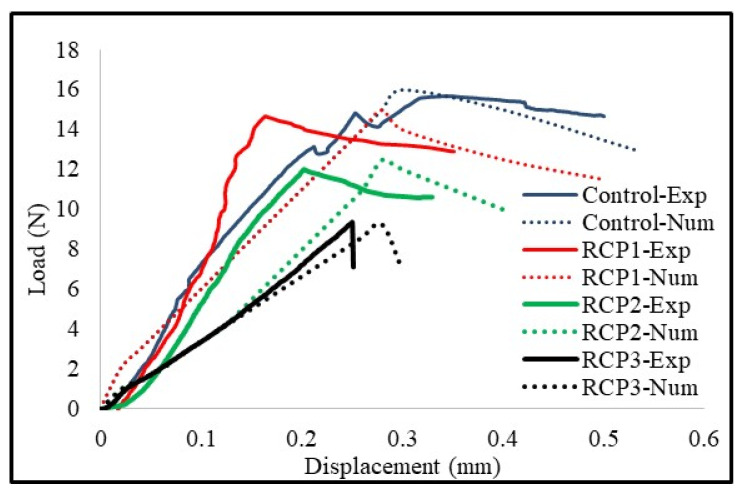
Numerical vs. experimental results of the four-point bending test of the beam.

Figure 23 presents the numerical analysis results calibrated against the experimental ones. The experimental results (out of three) were selected for those samples whose values were close to the average values of that group. The results of the numerical analysis align closely with the experimental findings. The output parameters of all specimens, precisely load and deflection, are well-matched. The load–deformation curve is a crucial tool for comparison, as it reveals insights into the initial stiffness, maximum load capacity, and post-peak response. All three of these parameters matched well, with no significant discrepancies observed in the overall response of the specimens. The difference between the peak load of the experimental and numerical samples was less than 10% for all samples, demonstrating the effectiveness and precision of the analysis. Moreover, there are many important factors that can be considered, like initial stiffness and maximum displacement as the governing factor taken as reference. However, considering the nature of testing (i.e., four-point bending) and the fact that plain concrete was used without any fibers or steel reinforcements, which makes it further brittle. Moreover, the maximum displacement of about 0.5 mm (Figure 23) further clarifies the fact. Thus, it was decided that the peak load should be taken as the governing factor, as it is more decisive in the current scenario.

Calibrating the model demonstrates its capability to simulate the concrete’s physical behavior under given loading and boundary conditions, providing confidence in its predictive power for various combinations of RCP as a partial replacement of cement. Moreover, the crack patterns and the type of failures also matched quite accurately, with flexural cracks initiating at the bottom middle of the beam that resulted in the beam failure. Thus, after satisfying the calibration conditions, a sensitivity analysis was designed to evaluate how RCP content influences the results and how it can be optimized based on the given boundary conditions and assumptions used in the modeling stage.

### Sensitivity Analysis

Successful validation of the numerical model provides credibility to the analysis and improves the understanding of how RCP and altered concrete strengths interact under load. Thus, sensitivity analysis is vital in assessing the impact of input variations, like concrete grade and RCP content, on overall performance. To make the analysis more effective and objective, only RCP content was varied in all three concrete mixes. The study considered a range of RCP proportions presented in Table 6 to optimize the RCP percentage.

**Figure 24:**
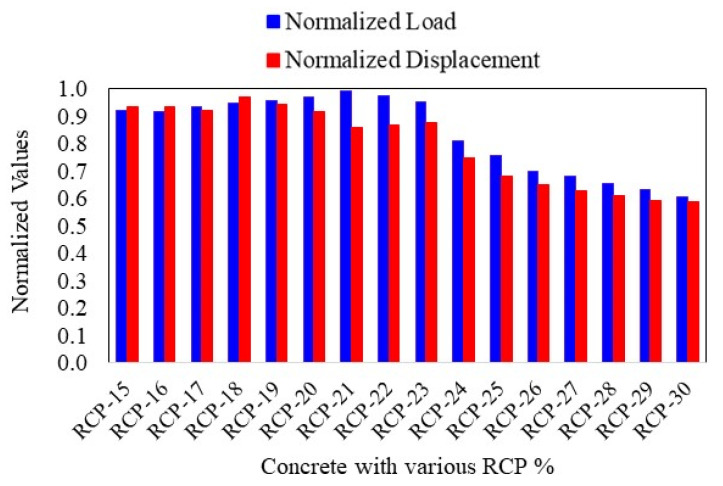
Results of the sensitivity analysis.

Figure 24 presents the sensitivity analysis results in normalized load and displacement. Since the sensitivity analysis aimed to optimize the RCP proportions, the normalized presentation of the results is the best approach to compare various parameters for the given samples, given that normalization was performed based on maximum values obtained for both results in all samples. The sensitivity analysis further strengthens the findings of the experimental study, i.e., RCP replacement up to 23% is the optimized value and can be used without compromising the mechanical properties of concrete. Although the current numerical study is limited to flexural strength and cannot be generalized, in combination with the experimental program, it can be concluded (with the above-mentioned limitations) that the overall mechanical properties of concrete do not degrade until 23% of the cement is replaced with the RCP material. Based on the sensitivity analysis, it can be concluded that 23% RCP replacement seems to be the optimized proportion for the maximum load and displacement in the four-point bending test. It should be noted that the results of this analysis are purely dependent on the boundary conditions fixed in the calibration study and the input parameters.

## 7. Conclusions

This study concentrates on the properties of sustainable concrete with various replacement amounts of RCP to enhance the sustainability of construction practices. Based on the investigation of the mechanical and microstructural characteristics of the examined samples, the following conclusions are found:

(1) As the replacement quantity of RCP increased, a corresponding reduction in slump was detected, signifying a decrease in workability for all the mixes. This conclusion proposes that larger replacement quantities harm the workability of the concrete.
(2) The suitable quantity of RCP can have the least impact on the strength of concrete at different curing ages. The best results were obtained for the RCP-1 mix (20% RCP contents), which had a 16.3% reduction in compressive strength at 7 days, a 16% reduction on 14 days, and a 20.7% reduction on 28 days as compared to the control mix. The reasons influencing the mechanical properties of concrete by RCP have been well examined and discussed. Nevertheless, the durability of concrete is yet to be considered under these conditions; the performance against fatigue and creep will also be considered in subsequent studies.
(3) In particular, the RCP-1 showed better performance, and various parameters used, e.g., split tensile strength, static elastic modulus, dynamic elastic modulus, rebound hammer test, ultrasonic pulse velocity test, etc., indicated the efficiency of RCP-1 in comparison with the control mix.
(4) With the SEM, it was revealed that in RCP-1, the shape and morphology are irregular, but the particle is larger, which may affect the hydration process and also reduce the workability of the mix. Moreover, the C-S-H gel between the particles is weak, affecting concrete performance. This pattern gets stronger as the amount of RCP is increased, and RCP-2 and RCP-3 depict weaker C-S-H gel and an excessive number of pores, resulting in permeability increment.
(5) In the case of RCP-2 (25% RCP contents) and RCP-3 (30% RCP contents), when the replacement rate increased from 20%, it started affecting the properties of concrete adversely, both at the fresh state as well as hardened state, indicating the rate to be kept around 20% maximum.
(6) The GWP study provides strong support for the use of RCP (recycled concrete powder) as a substitute for traditional cement. This replacement results in a substantial decrease in CO_2_ emissions associated with concrete production, making it a more environmentally friendly option. By integrating RCP into construction practices, we can promote sustainability in the industry and reduce the overall carbon footprint of building materials. This shift not only benefits the environment but also aligns with the growing demand for greener construction solutions.
(7) An optimization study performed through numerical modeling reveals that up to 23% replacement of cement with RCP can be performed without compromising the mechanical properties of concrete.

## Figures and Tables

**Table 1:** XRF (X-ray fluorescence) results comparison of cement and RCP.

Parameters	Cement	RCP
SiO_2_ (Silica)	20.63	22.55
Al_2_O_3_ (Aluminum)	4.94	5.90
Fe_2_O_3_ (Iron)	3.10	1.57
CaO (Calcium)	60.47	32.40
MgO (Magnesium)	3.17	2.40
SO_3_ (Sulfite)	2.52	1.94
Loss of Ignition	3.94	28.44

**Table 2:** Material properties for various mixes used in the numerical models.

No.	Properties	E (GPa)	G_F_	v	f_t_ (MPa)	f_c_ (MPa)
1	Control	24.69	62.85	0.2	2.60	28.8
2	RCP-1	23.28	45.96	0.2	1.86	18.01
3	RCP-2	21.20	33.68	0.2	1.35	11.30
4	RCP-3	19.82	30.75	0.2	1.23	9.89

**Table 3:** Modulus of elasticity of concrete by various proposed models in the literature.

Model Proposed by	Relationship	Elastic Modulus Values (ksi)			
		Normal	RCP-1	RCP-2	RCP-3
ACI-363.R [67]	$Ec=3320{\left(f^{\prime }c\right)}^{0.5}+6900$	3043.63	2819.76	2616.22	2514.50
Danha et al., 2013 [68]	$Ec=113.43f^{\prime }c+31,126.74$	4809.64	4748.27	4698.65	4676.06
Kadhem et al., 2018 [69]	$Ec=14,000{\left(f^{\prime }c\right)}^{0.25}$	4182.06	3946.30	3719.00	3600.03
CEB-FIP [70]	$Ec=22,000{\left(f^{\prime }c/10\right)}^{1/3}$	3881.36	3592.39	3319.18	3178.36
Noguchi et al., 2009 [71]	$Ec=k_{1}k_{2}33,500{\left(f^{\prime }c/60\right)}^{1/3}{\left(\rho /2400\right)}^{2}$	3165.67	2929.98	2707.15	2592.29

**Table 4:** UPV test results (average) for various mixes.

Specimen No	Pulse Velocity (km/s)
Control	4.64
RCP-1	4.52
RCP-2	4.24
RCP-3	4.35

**Table 5:** Ratio of E_c_ to E_d_ for various concrete mixes.

E_c_ Obtained by	Ec/E_d_			
	Normal	RCP-1	RCP-2	RCP-3
ACI-363.R [67]	0.47	0.45	0.48	0.44
Danha et al., 2013 [68]	0.74	0.76	0.86	0.82
Kadhem et al., 2018 [69]	0.64	0.63	0.68	0.63
CEB-FIP [70]	0.59	0.58	0.61	0.55
Noguchi et al., 2009 [71]	0.48	0.47	0.50	0.45

**Table 6:** Various fiber percentages considered in the sensitivity analysis.

No.	1	2	3	4	5	6	7	8	9	10	11	12	13	14	15	16
RCP Percentage	15%	16%	17%	18%	19%	20%	21%	22%	23%	24%	25%	26%	27%	28%	29%	30%

## Data Availability

The original contributions presented in this study are included in the article. Further inquiries can be directed to the corresponding author..
